# Applications of Artificial Intelligence in Screening, Diagnosis, Treatment, and Prognosis of Colorectal Cancer

**DOI:** 10.3390/curroncol29030146

**Published:** 2022-03-07

**Authors:** Hang Qiu, Shuhan Ding, Jianbo Liu, Liya Wang, Xiaodong Wang

**Affiliations:** 1 Big Data Research Center, University of Electronic Science and Technology of China, Chengdu 611731, China; hbigdata@uestc.edu.cn; 2 School of Computer Science and Engineering, University of Electronic Science and Technology of China, Chengdu 611731, China; 3 School of Electrical and Computer Engineering, Cornell University, Ithaca, NY 14853, USA; sd925@cornell.edu; 4 West China School of Medicine, Sichuan University, Chengdu 610041, China; liujianbo@stu.scu.edu.cn; 5 Department of Gastrointestinal Surgery, West China Hospital, Sichuan University, Chengdu 610041, China

**Keywords:** colorectal cancer, artificial intelligence, machine learning, deep learning, diagnosis, prognosis, treatment, screening

## Abstract

Colorectal cancer (CRC) is one of the most common cancers worldwide. Accurate early detection and diagnosis, comprehensive assessment of treatment response, and precise prediction of prognosis are essential to improve the patients’ survival rate. In recent years, due to the explosion of clinical and omics data, and groundbreaking research in machine learning, artificial intelligence (AI) has shown a great application potential in clinical field of CRC, providing new auxiliary approaches for clinicians to identify high-risk patients, select precise and personalized treatment plans, as well as to predict prognoses. This review comprehensively analyzes and summarizes the research progress and clinical application value of AI technologies in CRC screening, diagnosis, treatment, and prognosis, demonstrating the current status of the AI in the main clinical stages. The limitations, challenges, and future perspectives in the clinical implementation of AI are also discussed.

## 1. Introduction

Colorectal cancer (CRC) is the third most common cancer and the second leading cause of cancer death worldwide [1]. The World Health Organization has estimated that more than 1.9 million new cases and 935,000 deaths occurred in 2020, accounting for about one-tenth of all cancer cases and deaths [1]. Although significant improvement has been made in CRC healthcare, the global incidence and mortality rates continue to rise and are expected to increase by 15% to more than 2.2 million new cases and 1.1 million deaths by 2030 [2]. It is therefore of great significance to identify novel and effective strategies for early diagnosis, accurate treatment assessment and prognosis prediction of CRC, which are essential to increase the survival rate.

**Figure 1:**
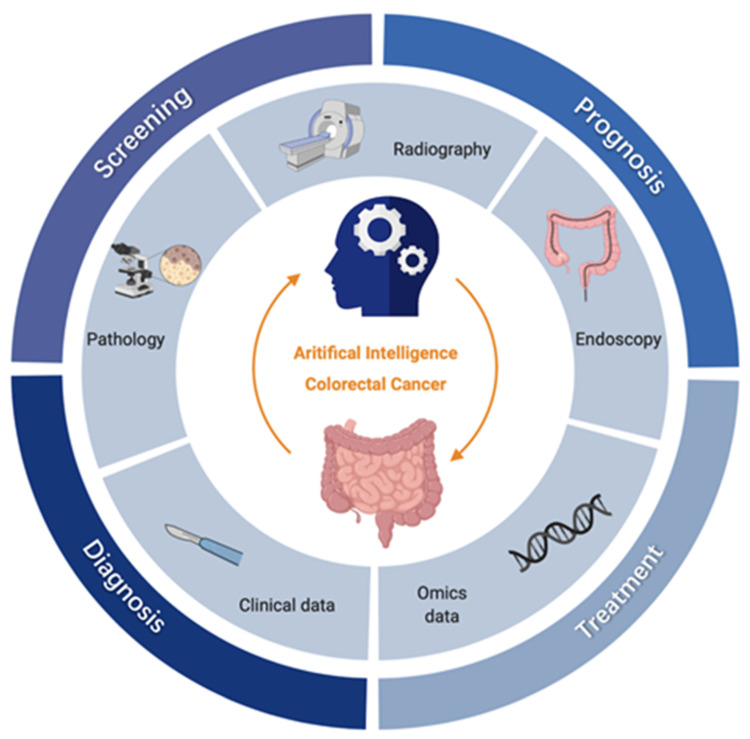
Clinical applications of AI for CRC. The inner circle represents the main data types in CRC research, including radiological images (i.e., Computed Tomography (CT), Magnetic Resonance Imaging (MRI), etc), endoscopic images, pathological images, clinical data, and omics data; the outer circle represents the four key clinical parts of CRC, i.e., screening, diagnosis, treatment, and prognosis; for each clinical part, AI has subdivided and specific tasks, which are shown in boxes outside the circle respectively. OS, overall survival; DFS, disease-free survival; nCRT, neoadjuvant radiotherapy.

Artificial intelligence (AI) technologies, especially machine learning (ML) and deep learning (DL), have advanced rapidly in medical care, providing new potential to build powerful and accurate computer-assisted methods that can effectively screen, diagnose, and treat cancer, and follow patient prognosis [3]. A large number of recent studies have applied AI in the field of CRC [4,5,6]. From the perspective of clinical practice, the existing applications of AI in CRC mainly involve four clinical parts (Figure 1):

- Screening: Endoscopy is considered the gold standard for CRC screening, supplemented with fecal occult blood test (FOBT), but these methods are relatively dependent on clinical experience and prone to omission and misdiagnosis. The increasing prevalence of endoscopic imaging datasets and electronic medical records (EMRs), AI-assisted endoscopy for polyp detection and characterization, and the use of high-risk prediction models using clinical and omics data, are expected to improve the accuracy and efficiency of CRC screening.
- Diagnosis: The qualitative diagnosis and staging of CRC mainly rely on radiography and pathological examination [5]. Thanks to advanced processing technology in the field of image recognition, DL can significantly improve medical image readability, eliminate differences in experience, and reduce misdiagnosis rates.
- Treatment: The most commonly used methods for clinical treatment of CRC are surgery, chemotherapy and radiotherapy [7]. Novel therapies and tools can be evaluated with the help of AI, such as neoadjuvant radiotherapy (nCRT) and chemotherapy, to improve curative effects and provide more precise medical care to patients.
- Prognosis: Prognosis of CRC includes the predicting of recurrence and estimating of the survival period [3]. Statistical methods such as the Cox regression model are traditionally used to predict patient prognosis; however, data-driven ML approaches allow for more effective exploitation of multidimensional data to accurately predict survival and flexibly track disease progression.

In this review, we comprehensively analyze and summarize the research progress and clinical value of AI technologies in the screening, diagnosis, treatment, and prognosis of CRC, providing a complete picture of the current status of the AI in the main clinical parts. We also discuss the limitations and challenges in the clinical implementation of AI and describe the efforts needed to resolve these issues. We hope that this information is beneficial to both clinicians and researchers interested in the applications of AI in the clinical care of CRC.

## 2. Overview of Artificial Intelligence

The concept of AI was first introduced in the 1950s [8], and it has continued to develop rapidly into the 21st century. The AI boom has also advanced medical fields, thanks to the technical support of infrastructure hardware and the continuous development of databases [9]. This section describes the basic concepts of AI, ML, and DL and focuses on CRC specifically in terms of common algorithms and available data types.

### 2.1. Basics Concepts of AI

**Figure 2:**
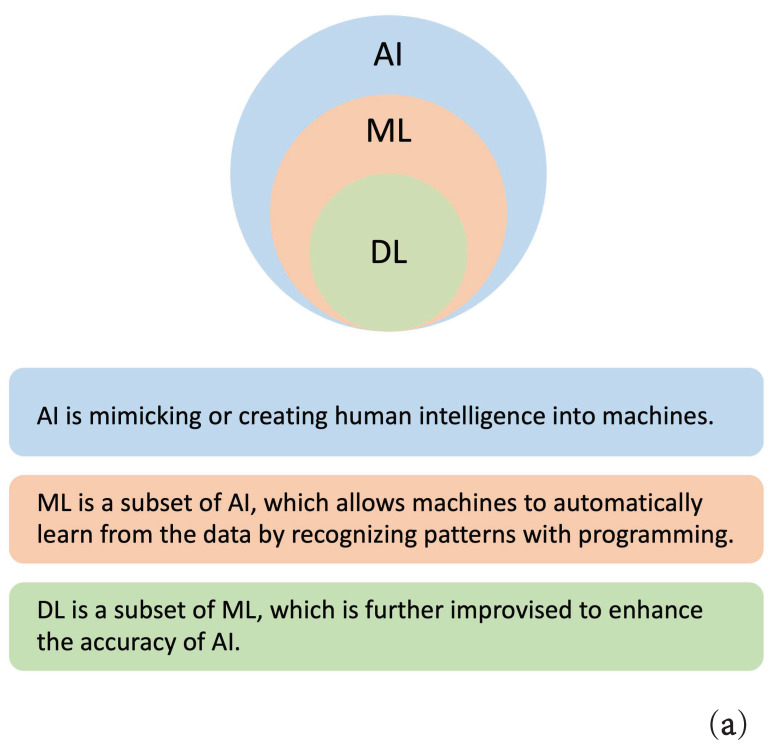

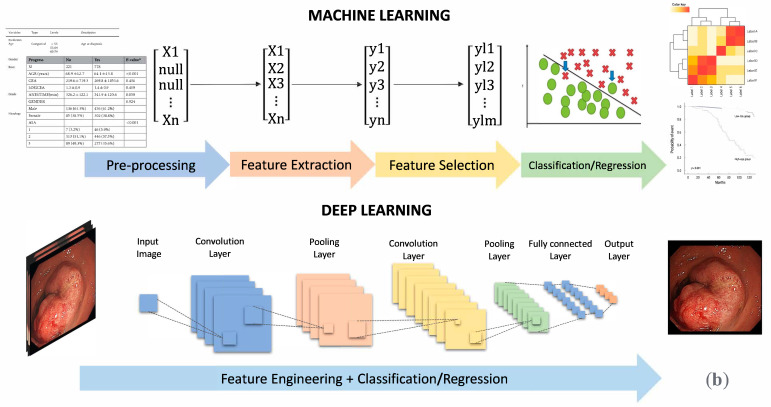
Basics concepts of AI, ML, and DL. (**a**) The relationships of AI, ML, and DL; (**b**) The workflows of ML and DL.

AI focuses on exploiting calculation techniques with advanced investigative and prognostic facilities to process all data types, which allows for decision-making that mimics human intelligence [10]. ML is a subfield of AI that enables a machine to become more effective with training experience. The principal learning models are mainly supervised learning, unsupervised learning, and reinforcement learning [3,11]. Semi-supervised learning is also gaining importance; this combination of supervised learning and unsupervised learning allows the use of both unlabeled data and labeled data. Models differ depending on the input data type and require various algorithms. Typical algorithms include Logistic Regression (LR), Support Vector Machine (SVM), Naive Bayes (NB), Gradient Boosting (GB), classification trees, and Random Forest (RF). Accruing massive amounts of data led to the development of DL, a subset of ML. DL technologies offer more intelligent computational networks and better predictive power by developing multiple layers of artificial neurons. The available Neural Network (NN) approaches in CRC research include convolutional NN (CNN), and recurrent NN (RNN). DL methods are widely employed for medical image classification, image quality improvement, and segmentation. The basic concepts and relationships of AI, ML, and DL are shown in Figure 2a.

The basic ML and DL workflows are summarized in Figure 2b. The ML process can be roughly divided into four steps: data pre-processing, feature extraction, feature selection, and classification/regression. DL processing merges these four steps into feature engineering and classification/regression. The critical difference lies in their understanding of features; in ML, the feature extraction is done manually by humans, while in DL, AI automatically generates a variety of features [6].

### 2.2. Data Modality

#### 2.2.1. Image Data

**Figure 3:**
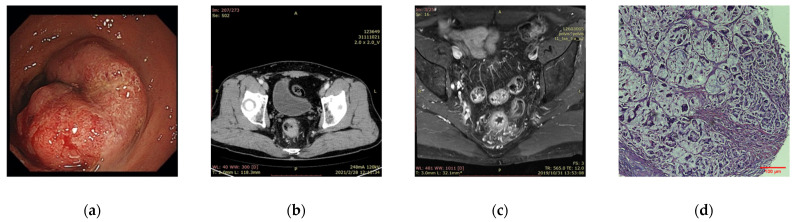
Common CRC image types. (**a**) Endoscopy; (**b**) CT; (**c**) MRI; (**d**) pathology image (the Hematoxylin & Eosin (HE)-stained slide). (Image courtesy: West China Hospital, Sichuan University).

The key developments and enhancements of AI in CRC have been widely applied in medical imaging. Relevant information can be extracted from various imaging data to enable tumor segmentation, feature extraction, and model building, and finally quantitative tumor evaluation. In CRC studies, we mainly focus on these four common types (Figure 3).

Image analysis is enhanced by the use of highly specialized algorithms. Detecting suspicious polyps and distinguishing between the normal and abnormal are key components of accurate diagnosis, which is especially important for early detection of CRC and improves patient prognosis.

#### 2.2.2. Clinical Data

Clinical data are advantageous for identifying high-risk CRCs and predicting treatment outcomes and prognoses. Because they are widely collected and accessible, clinical data constitute a significant database in CRC. For AI applications, useful features are captured from three aspects: (1) patient demographic information (e.g., age, sex, race, and smoking and drinking history); (2) laboratory test results (e.g., complete blood count (CBC), carcinoembryonic antigen level); and (3) histopathologic information: (e.g., cancer and tumor-related information such as location, tumor size, stage, margins).

EMR systems have the potential to capture large sets of clinical data relating to hospital visits, medical history, lab and pathology results, prescriptions, and social and demographic information [12]. With the increasing adoption of these systems come greater possibilities for utilizing this data to improve patient outcomes.

#### 2.2.3. Omics Data

“Precision medicine” is gaining momentum in medical AI applications. The analysis of CRC omics data shows tumor pathogenesis at the molecular level, thus providing a reliable reference for targeted therapy and genome sequencing, leading to improvements in CRC diagnosis and prevention [13]. There are three main omics data in CRC: (1) genomics data can elucidate CRC pathogenesis and provide targets and evidence for targeted treatment [14]; (2) proteomics data can identify proteins associated with CRC which have the potential to provide biomarkers for CRC screening and early diagnosis of CRC [14]; and (3) metabolomic data of tissues, blood, and urine indicate that patients with different stages of CRC present with multiple metabolic pathway abnormalities involving multiple biochemical reactions [13].

Although there are cost and facility constraints that prevent the widespread use of omics data, it has been shown to be highly valuable in CRC research settings. The utilization of ML and DL techniques based on large-scale omics data enables active research and is a novel medical approach to identifying the best treatment options for CRC patients.

## 3. Applications in CRC Screening

Screening is intended to effectively reduce CRC incidence and mortality, facilitate early diagnosis and treatment, and thus improve patient prognosis [15]. Endoscopy and FOBT are routine screening methods, but they have limitations. With applications of AI technology in the field of tumor screening, many new CRC screening prediction models, techniques, and potential biomarkers have emerged that are expected to improve the accuracy and reduce the cost of CRC screening. A summary of recent studies on AI applications for CRC screening is presented in Table 1.

### 3.1. Polyp Detection and Characterization

Endoscopy is the most effective screening tool available today [33]. However, a systematic review showed that the miss rate of endoscopy for any size polyp was 22% and this significantly increased for smaller lesions [34]. In addition, the combination of endoscopy, a CRC-specific screening method, and AI is rarely addressed in other related cancer studies. Given the excellent processing and analysis capabilities for complex images, AI-assisted endoscopy has the advantages of improving the ability to detect and characterize polyps, minimizes trauma to patients, and eliminates variation due to different clinicians.

The main focuses are on the detection [20,22,23,24,30] and classification of polyps [17,27,32]. Chen et al. [35] applied a deep NN (DNN) to narrow-band imaging endoscopy to automatically identify hyperplastic or neoplastic polyps under 5 mm. A total of 1476 neoplastic and 681 hyperplastic polyp images from a tertiary Taiwan hospital were collected to train the DNN, and a new cohort (96 hyperplastic and 188 neoplastic polyp images) was utilized for testing. The results showed that this system achieved a sensitivity of 96.3%, specificity of 78.1%, positive predictive value of 89.6%, negative predictive value of 91.5%, and reduced the image reading time by 0.45 ± 0.07 s compared to endoscopists. This DL model yielded excellent classification results and reduced the time required for examination, improving screening accuracy and efficiency. In addition, for non-polypoid lesions, Yamada et al. [36] developed a real-time AI diagnostic system that automatically detects early signs of CRC during endoscopy; the sensitivity and specificity of the system were 97.3% and 99.0%, respectively, and the area under the curve (AUC) was 0.975. This AI system notified endoscopists in real time to avoid missed diagnoses such as non-polypoid polyps. It is expected to bridge the gap in diagnostic quality among different clinician levels and improve early CRC detection.

There is also research about the localization and segmentation of polyps [21,25]. Akbari et al. [25] proposed a CNN-based method for polyp segmentation. The CVC-ColonDB database was used to evaluate the results and their model achieved a specificity of 74.8%, sensitivity of 99.3%, and accuracy of 97.7%, thus achieving accurate region-of-interest segmentation and providing a basis for subsequent processing.

### 3.2. Population-Based Risk Prediction

Large-scale, population-based data can be utilized to identify high-risk populations and develop preventive intervention strategies for CRC. AI can assess the risk of CRC for a broad population based on demographic data [26], blood/stool tests [18,19,28,29,31], and omics data [16,37].

Complete Blood Count (CBC) laboratory test is a relatively new screening method to help identify high-risk patients [28,31]. Kinar et al. [31] conducted a binational study between Israel and UK to develop an ML-based prediction model (MeScore) for identifying individuals at high risk of CRC based on EMRs. The AUCs for detecting CRC were 0.82 ± 0.01 and 0.81 for the Israeli and UK validation sets, respectively. When FOBT was also taken into consideration, the number of new cases increased by 115%. This study showed that MeScore could detect high-risk patients in a primary care setting and potentially decrease the risk of developing CRC. Hornbrook et al. [28] similarly proposed an AI system that predicted early CRC by analyzing patient information, including gender, age, and CBC data. This system provided a reference for whether an individual should undergo endoscopy. As this system continues to improve, it is expected to be useful for exploring important indicators for CRC diagnosis.

Serum biomarkers, such as N-glycans [18] and protein biomarkers can also provide an efficient screening method for early CRC [19]. Ivancic et al. [19] investigated the utility of mass spectrometry-based serum protein biomarker assays for screening for CRC. They collected blood samples from individuals (*n* = 213) and non-metastatic CRC patients (*n* = 50). ML models such as LR and SVM were used to make predictions. Peptides from EGFR (Epidermal Growth Factor Receptor) and LRG1 (Leucine Rich Alpha-2-Glycoprotein 1) were consistently identified as highly predictive. LRG1, EGFR, ITIH4 (Inter-Alpha-Trypsin Inhibitor Heavy Chain Family Member 4), HPX (Hemopexin), and SOD3 (Superoxide Dismutase 3) formed the best performing group with 70% specificity and over 89% sensitivity (AUC = 0.86). On the other hand, Pan et al. [18] selected an N-glycan-based serum biomarker to identify for screening and diagnosis of advanced adenomas and CRC. They used ML models, including RF, LMT, and SVM, to classify 189 samples from CRC, advanced adenomas, and healthy controls, obtaining an accuracy of 75% for the whole group and 87% for the disease group (CRC and advanced adenomas). The minimally invasive blood biomarker approach has valuable potential as an alternative method for CRC screening.

Sequencing the CRC genome can improve understanding of tumor pathogenesis at the molecular level, thus enhancing the early detection of CRC [38]. Wan et al. [37] applied ML methods for whole-genome sequencing of plasma cell-free DNA to detect early CRC. They extracted reads aligned to protein-coding gene bodies from 546 patients with CRC and 271 non-cancer controls. The results yielded high accuracy (AUC = 0.92) and great sensitivity and specificity, especially in early CRC cohorts.

### 3.3. Limitations

Overall, screening with AI technologies is likely to increase the detection rate of clinically relevant polyps which may be precancerous lesions [26,27,34] and biomarkers to prevent CRC [33,34,36]. However, it may also increase the overdiagnosis of early stage cancers that have no malignant potential, enhancing patient suffering and wasting medical resources [39]. Most previous studies focused on how to improve the polyp detection rate and the accuracy of predicting early CRC [20], which is prone to overdiagnosis. In addition, it is difficult to access long-term follow-up results, so the benefits and harms of AI in CRC screening cannot be accurately evaluated.

## 4. Applications in CRC Diagnosis and Staging

Diagnosis of CRC includes qualitative and staging diagnosis, determining whether a patient has the CRC pathologically, and an assessment of the severity of the tumor [40]. The current qualitative diagnostic approach for CRC includes biopsy collection during endoscopy or surgery followed by histopathology. The staging diagnosis mainly relies on radiological examinations, such as CT, MRI, etc. The incorporation of AI technology is intended to help clinicians improve diagnostic efficiency, reduce workload, and improve medical image readability, ultimately reducing the rates of misdiagnosis and missed diagnoses. Table 2 presents a summary of recent studies on AI applications for CRC diagnosis and staging.

### 4.1. Pathological Diagnosis

As the “gold standard” for tumor diagnosis, pathology characterizes the disease before surgery and serves as the basis for postoperative CRC staging [64]. However, biopsy sample diagnoses can be easily biased by individual pathologists’ experience and knowledge, leading to inter- and intra-observer variations. Current applications of AI technology in pathological diagnosis are mainly focused on gland segmentation and tumor classification.

For gland segmentation, Ding et al. [65] showed the state-of-the-art. They developed a three-class classification multi-scale fully convolutional network model (TCC-MSFCN) with 165 histological images of Hematoxylin & Eosin (HE)-stained slides with associated intensive ground truths. The model achieved better performance by combining features such as dilated convolution, high-resolution branch, and residual structure. The foremost advantage is that the MSFCN model can also segment the glands with a variable dataset, and TCC can precisely differentiate very closely spaced glands. The authors also performed a series of experiments with a variable dataset to check the robustness of their combined features.

Several ML models have been developed to reduce the variation in classifying tumors into various subtypes [41,42,45,46,47,48,49,50,51,66]. Rathore et al. [66] developed a novel CRC detection system based on the SVM radial basis function algorithm, which classified normal colon biopsy images and malignant images, and then automatically determined malignancy grades. Compared to previous techniques, this system showed superior cancer detection (accuracy 95.40%) and grading (accuracy 93.47%) capabilities. Based on this system, the same team subsequently proposed a hybrid feature-space-based colon classification (HFS-CC) technique that classified biopsy sample images using multiple features, including geometric features, morphology, and texture. An SVM was used as a classification tool to classify 176 subjects, and the HFS-CC technique achieved a test accuracy of 98.07%.

Furthermore, some models achieved both gland segmentation and classification in two stages. Xu et al. [50] proposed a CNN-based approach for gland segmentation and classification in benign and malignant CRC tissues. The system constructed two CNNs for pixel-level classification of HE-stained images. The first classifier separated the glands from the background and the second classifier identified the gland separation structure. The results showed that 98% and 95% accuracies were achieved in distinguishing benign and malignant tissues, respectively. Moreover, Takamatsu et al. [67] also performed a prediction of lymph node metastasis with immunohistochemistry images. Several morphological parameters were extracted from 397 T1 CRC immunohistochemistry images and then RF was applied to train and test. Comparing their results with HE-stained slides, there were no significant differences and even fewer false-negative cases. This suggests that immunoimaging is a potential alternative to T1 CRC diagnosis.

In terms of immunotherapy, current research is also mainly in diagnosis. In particular, immunotherapy usually requires precise target selection as a basis, and genetic testing is one of the important tools used to determine the target. Existing research attempts to use pathological images combined with genes for analysis. Ge et al. [44] used a deconvolution algorithm, CIBERSORT, to analyze the infiltration of 22 immune cell types in the tumor microenvironment and the expression of immune-related genes in 404 cases of CRC and 40 cases of adjacent non-tumor tissues. Such research results have further optimized gene-based and individualized diagnosis methods, which are conducive to providing support for future targeted therapies and immunotherapy. However, in-depth research on the impact of diagnosis on treatment and the prognosis after treatment is still lacking.

### 4.2. Radiological Diagnosis

Since the concept of “radiomics” was introduced, it has become a hot spot for clinical research [68]. Radiomics extracts information from various imaging data, and finally realizes quantitative evaluation of CRC through tumor segmentation, feature extraction, and model development. DL technology, which is widely used in the field of image recognition, can significantly improve medical image readability and provide reliable and comprehensive references for clinicians.

CT is a rapid non-invasive radiography test to detect polyps [59,60,61,62,63] and results in a lower risk of adverse effects than endoscopy [69]. Taylor et al. [63] utilized a computer-aided detection (CAD) system to assess the ability to detect flat polyps on CT images. Endoscopic reports annotated by joint experts were considered as the standard, in which 24 stage T1 patients were classified as type IIa (*n* = 11) and Iia + Iic (*n* = 13). The results showed that >96.1% of true positives on CAD were classified as lesions, supporting the use of this CAD system to detect flat polyps.

Moreover, Khalili et al. [70] used CT scan images to detect small hypoattenuating hepatic nodules (SHHN) in CRC with CNNs. They constructed supervised learning CNNs and a multivariate model, which was for compensating other representations. Results were presented as the Receiver Operating Characteristic (ROC) and Area Under the ROC Curve (AUC) and compared with three radiologists. Radiologists outperformed CNNs in classifying SHHN as benign or malignant (ROC = 0.96, ROC = 0.84 respectively), but were comparable to CNNs adjusted for multivariate modeling. CNN combined with liver metastasis status was almost equivalent to expert radiologists’ diagnostic accuracy but with better diagnostic confidence.

González-Castro et al. [56] utilized the Haralick texture feature of CT scan images to detect KRAS mutations, classified as KRAS+ and KRAS-. This was achieved by four ML algorithms, SVM, Gradient Boosting Machine (GBM), NN, and RF, where the wavelet transformed and Haralick coefficients were used as the feature vector for the NN classifier, resulting in the highest accuracy and kappa values of 83% and 64.7%, respectively. It is an advance to identify gene mutations directly from CT, as it avoids the diverse effects caused by invasive testing; this approach also prevents errors from biopsying only part of the tumor and provides more personalized and effective treatment.

MRI is the preferred modality for assessing tumor location and lymph node metastasis, which is a key indicator to assess tumor severity. Lu et al. [71] developed a model of lymph node metastases using Faster R-CNN based on 28,080 MRI images and performed multicenter clinical validation among 414 patients across six medical institutions. The results indicated that the system had an AUC of 0.912, which was clinically feasible, and it took 1/30 of the time needed by radiologists. The Faster R-CNN algorithm is very efficient and accurate in predicting lymph node metastases, which reduces the workload on the radiologist and minimizes differences between different diagnostic levels. Some other representative research about lymph node metastasis are listed in Table 2 [52,53,54,55,57,58,72].

### 4.3. Limitations

Most applications in CRC diagnosis have focused on radiological and pathological images [64,73,74,75]. The advanced image processing capabilities of AI could assist clinicians in decision making and reduce unnecessary variation between clinicians with varying expertise [76]. CNN is the most widely used method for CRC diagnosis, however, due to the “black-box” nature of DL, medical interpretability is difficult guarantee [77]. Moreover, the method and quality of image acquisition largely influences decisions, a standardized public image database will be needed in the future. At the same time, there is a relative lack of research using clinical data for AI diagnosis. In addition, because targeted therapies and immunotherapy are still in ongoing clinical research, there are few AI studies related to these treatments.

## 5. Applications in CRC Treatment

Treatment options for CRC include nCRT, chemotherapy, and other comprehensive approaches [78]. Applying AI technology to CRC treatment can help clinicians choose the appropriate treatment options for patients and improve treatment efficacy by designing personalized and precise treatment plans. There is some integration with AI technology focusing on the prediction of nCRT and chemotherapy response. Table 3 provides a summary of recent studies on AI applications for CRC treatment.

### 5.1. nCRT Response Prediction

nCRT is of great clinical importance for patients with CRC, especially those with rectal cancer [7]. It provides a chance of achieving pathological complete response (pCR), which is associated with a good prognosis and may preclude the need for surgery [80]. Therefore, if the effect of nCRT can be predicted in advance, it will better help clinicians to select an appropriate treatment plan.

Current nCRT research is based on two considerations, basic clinical data [79,80], and radiology images [81,82]. Tan et al. [80] performed pCR prediction based on demographics and tumor characteristics in patients with non-metastatic rectal cancer who underwent radical resection after nCRT. They used the LR to determine independent predictors of pCR and showed that clinical T4 and N2 stages were the most important independent clinical predictors. The 3-year overall survival rates of patient with and without pCR were 92.4% and 88.2%, respectively. Ferrari et al. [82] used RF to construct two models based on texture analysis of high-resolution T2 weighted MRI to predict the pCR and pathological non-response (pNR) rates, and the efficiencies of these two systems for identifying the two types of cases were 0.86 and 0.83 respectively. This study screened pCR and pNR cases after nCRT, thus providing a guarantee for partial resection or observational treatment for pCR patients and the selection of further appropriate treatment for pNR patients.

### 5.2. Adjuvant Chemotherapy Response Prediction

Accurate AI prediction of the chemotherapy response provides better personalized medicine options for patients and improves the survival rate [85].

Irinotecan (CPT-11) is a commonly used in chemotherapy drug for CRC. However, due to its high adverse reactions, the effect–risk ratio in adjuvant chemotherapy for patients is too low. Oyaga-Iriarte et al. [83] developed an ML model to predict the toxicity of CPT-11. Their study collected basic information and serum biomarker levels at different stages from 20 patients and constructed an SVM model based on the irinotecan levels and metabolites. The prediction accuracy of the algorithm was 91% for diarrhea, 76% for leukopenia, and 75% for neutropenia, which could provide a reference for clinicians’ decisions. In addition, from the current research reports, there is no similar AI research on the effects and risks of oxaliplatin.

AI technology also contributes to new drug research. Cruz et al. [84] detected the half maximal inhibitory concentration (IC50) of a new drug targeting the HCT116 cell line. The Quantitative Structure–Activity Relationship (QSAR) was assessed using molecular and Nuclear Magnetic Resonance (NMR) descriptors based on KNN, RF, and SVM techniques. This NMR QSAR classification model achieved an overall prediction accuracy of over 63%. Their method provided support for the development of new drugs to treat CRC.

### 5.3. Limitations

There is limited research on the application of AI in CRC treatment, most studies have assessed algorithms’ ability to predict the response after nCRT and chemotherapy. The sample sizes of these studies are small and therefore lack good generalization performance [9]. For larger samples, AI research on common chemotherapy regimens based on guidelines is still lacking [86,87]. It should be noted that surgery is the most important treatment for CRC, although how its therapeutic effects and risks can be predicted through AI, is yet to be determined. Moreover, the combination of data transformed by surgical technology and AI technology is likely to become the unique feature of AI technology’s application in CRC. The surgical techniques of CRC not only brings about the clinical treatment effect, but may also affect the quality of life of the patient. For example, the surgical technique of rectal cancer takes into account the patient’s subjective appeal of preserving the anus. However, AI can also provide personalized treatment options by matching similar patients’ treatment modalities with past data. This is an area where we hope AI could be greater utilized in CRC treatment.

## 6. Applications in CRC Prognosis

CRC prognosis involves the recurrence and survival of patients [9]. Traditional statistical analysis cannot provide desired prognosis effect and it is difficult to predict the progress of patients. However, AI can process and analyze significant features based on previous data to predict cancer prognosis more accurately, as well as patient survival time and disease progression. Table 4 demonstrates the detailed information of recent research studies on CRC prognosis.

### 6.1. Recurrence Prediction

Estimation of recurrence is integral to patient management and forms the basis of cancer staging and treatment planning [92]. Historical data can be used to build predictive models that try to identify the relationship among patient characteristics.

Patients at high risk of recurrence after undergoing curative (R0) resection for CRC may benefit most from adjuvant therapy and follow-up for early detection and recurrence treatment [88,89]. Weiser et al. [92] developed a nomogram for predicting recurrence after R0 surgery, based on a dataset of non-metastatic CRC patients. The recurrence nomogram allowed for better consideration of tumor and patient heterogeneity, thus providing more personalized prognoses of outcomes.

Besides methods that rely on manual feature extraction, some studies use DL to optimize feature selection [90,91]. Li et al. [90] integrated CNN models into a proportional risk model to improve image features and build a survival regression model. Positron Emission Tomography (PET)-CT imaging data of patients with advanced rectal cancer were used to learn informative features to predict the time of local tumor recurrence. The results showed that the model had better predictive ability compared with the survival prediction models of the Cox proportional hazard model and random survival forest model (c-index = 0.60, 0.58, and 0.64, respectively).

In addition to predicting short-term post-operative recurrence, Joensuu et al. [91] established a long-term risk stratification scheme (10 and 15 years). The risk of recurrence was accurately predicted using a nonlinear model (AUC = 0.88, 0.86–0.90), as well as independent prognostic factors were sorted including tumor size, high mitotic count, non-gastric location, presence of rupture, and sex. Risk stratification schemes can help us identify patients who may be cured by surgery alone.

Recurrence prediction focusing on a specific stage is also required. Although existing staging systems such as the American Joint Committee on Cancer (AJCC) are easy to implement, there is significant heterogeneity within each group, so a refined method is needed. Takenaka et al. [88] focused on stage II CRC, evaluating recurrence-free survival (RFS) as the primary outcome. Proportional hazards models were statistically analyzed to identify factors associated with RFS and a nomogram was developed to depict the results. Xu et al. [89] predicted the feasibility of post-operative recurrence risk in stage IV CRC patients and ranked important influencing factors. They compared four basic ML algorithms and showed that GB and LightGBM outperformed LR and DT.

### 6.2. Survival Prediction

Survival predictive models allow clinicians to evaluate CRC prognosis and make patient-individualized choices for interventions. Current state-of-the-art analytic methods in CRC prognosis for survival analysis are statistical approaches [3], but they are not well-suited to handle large volumes of data or identify complex relationships between variables. AI may be able to utilize this data more effectively to better estimate patient viability and survival time.

CRC prognosis is highly dependent on pathology [95,96,98,102]. Kather et al. [96] automatically extracted prognostic factors based on HE-stained CRC tissues using CNN. This study performed tissue disaggregation of 862 HE slides collected from 500 stage I-IV CRC patients and achieved a classification accuracy of over 94%. The authors demonstrated that CNN could assess the tumor microenvironment and predict prognosis directly from histopathology images as well as detect additional prognostic markers on pathology sections. Similarly, Bychkov et al. [102] also employed pathology images to predict patient outcomes. Their model was based on a hybrid network, with a pre-trained CNN to extract feature vectors, and a recurrent neural network (LSTM) to read the CNN sequences to predict survivorship. However, they did not classify the intermediate tissues like Kather et al. [96]; rather, they directly used 420 digitized HE-stained samples to predict the 5-year CRC-specific survival of patients with an AUC of 0.69, showing an expert-level accuracy. Although their model was simpler and achieves equally good performance, additional validation is required due to the difficulty of interpreting the intermediate process and the small sample size.

Based on clinical data, we can find the relationships between patient characteristics and survival, allowing for a more precise prediction [94,97,99,100,101,103,104]. Sailer et al. [104] compared 10 common data mining algorithms to predict the binary target of 5-year survival based on seven attributes (sex, Union for International Cancer Control stage, etc.). The average accuracy of the ML was 67.7%, which was slightly higher than that of clinicians’ judgment, 59%.

Notably, there is also research that not only predicted the survival of patients but also regressed the remaining life span. Wang et al. [97] constructed a two-stage tree model with the Surveillance, Epidemiology, and End Results (SEER) Program dataset; in the first stage, a tree model based on unbalanced samples was proposed that predicted whether patients survived >5 years; in the second stage, data from group with <5 years’ survival were regressed by a selective ensemble model to predict the specific number of survival months. The results showed that the proposed two-stage model achieved more accurate predictions compared to the single-stage regression model.

### 6.3. Limitations

Current prognostic research focuses on linking clinical features to prognostic status through AI algorithms, resulting in highly accurate prognostic prediction systems that provide clinicians with diagnostic and treatment advice [93]. However, there are still significant differences in the sensitivities, specificities, and accuracies of the relevant AI technology applications, and most studies have been retrospective, so open prospective investigations are needed [31,99,105].

## 7. Current Challenges

While AI technology is rapidly being incorporated into clinical CRC research, the application of AI in CRC is still in its infancy compared to other oncology fields such as lung cancer and breast cancer [13]. Several challenges need to be addressed to translate these studies into clinically meaningful applications.

Generalizability of the AI algorithms is one of the biggest barriers preventing their widespread clinical adaptation. The predictive models with high sensitivity, specificity, and accuracy constructed by AI algorithms need to be based on a large amount of high-quality clinical data, so standardized data annotation and multicenter data sources are highly desired. To date, most of the AI algorithms in CRC are confined to data from one single medical institution [100], which may lead to model over-fitting and models that are not fully applicable in a broader context, especially for large and heterogeneous populations of CRC patients. Thus, external validation is necessary prior to widespread clinical adaptation of the AI applications.

In addition, interpretability is an important consideration for AI applications in CRC. Although DL models showed excellent accuracy in the diagnosis, and prognosis of CRC [90], they are considered “black boxes” due to their lack of interpretability. This issue is currently addressed in two main ways [106]. One is the interpretable model, such as linear models or DT. Most CRC research focuses on traditional statistical ML methods [12], and the results can be understood as a series of choices made based on features. The second involves model-independent interpretation methods, such as partial dependence plots [77], and the surrogate Mmodel. Visualization or a modular interpretation approach explains the internal working mechanism of predictive models, but this comes at the extra expense of some computational complexity and increased cost. There is still much to be done to improve model interpretability.

Moreover, most existing studies on the applications of AI in CRC were designed retrospectively [96,105]. Although the results from these studies appeared to be promising, solid evidence on the effectiveness of AI applied in CRC is still lacking. Due to potential selection bias in the retrospective study design, further prospective and multicenter investigations are required to confirm the utility of AI in clinical practice of CRC.

Furthermore, the safe management and use of clinical data are also important challenges. Compared with other research fields, establishing of AI applications for CRC requires a large amount of clinical data from patients, which requires privacy protection and raises ethical issues. There is ongoing research to address these problems; for example, Li et al. [107] proposed a multicenter RF prognostic prediction model that performed with desirable predictive capability and provided privacy guarantees. Drawing on such methods, it is expected that secure and reliable multicenter data sharing platforms for CRC can be established.

## 8. Future Prospects

Given the current status of AI in CRC clinical applications, we believe that future research in screening, diagnosis, treatment and prognosis will be directed as follows:

In the screening of CRC, the current gold standard is endoscopy and pathological biopsy [33]. In future, the research direction of AI technology should be focused on less-invasive technology compared to colonoscopy, while the diagnostic accuracy must remain close to pathological biopsy, or improve the diagnosis of precancerous lesions. Future research should first choose to use clinically apparent data, that is, the patient’s health status, disease history, symptoms that may seem unrelated, and treatment records before the discovery of CRC. Using these clinical data, combined with the information of the first diagnosis of CRC, AI technology can be used to establish a model of comorbid characteristics to help healthy people early warning of the occurrence of CRC.

Aiming at the diagnosis of CRC, AI technology should focus on the accuracy of TNM staging [108]. In other words, we need to establish a more accurate AI prediction system, not only for the T, N, and M staging, but also the overall preoperative staging status and the high-risk factors for recurrence in CRC patients before surgery. Through the establishment of preoperative clinical AI-TNM staging, accurate decision-making on the choice of neoadjuvant therapy and the formulation of surgical plans is possible. The consideration of this clinical staging requires a breakthrough in pure imaging technology, and comprehensive proteomics, metabolomics, genetic, and clinical epidemiological data should be considered.

However, the research of AI technology in the treatment of CRC still lacks breakthrough progress. The key point is how to closely integrate AI with surgical treatment. This close integration does not refer to AI technology-related operating methods, such as the application of robotic technology [109], but rather to the evaluation of the effect of AI technology on the surgical process, evaluation of the difficulty, and judgment of the quality of the operation. Because surgery is the most important link in the treatment of CRC, and it has been the technology of surgeons since the very beginning, how to associate AI with surgical technology is the key to the application of AI technology in CRC treatment.

For prediction of the prognosis of CRC, the AI technology needs to expand the application of the overall sample size and cross-crowd ethnographic database. A sufficiently large CRC database is sufficient to support the prediction of prognosis by AI technology [107], and it can help clinicians to find the factors that have the greatest impact on the prognosis, so as to establish future prospective prognostic intervention research. At the same time, for important clinical events such as CRC recurrence and metastasis, AI technology can also be used to predict serious oncology changes, which is also a valuable clinical breakthrough.

## 9. Conclusions

Due to the explosion of clinical data, and groundbreaking research in ML, and especially DL, AI has shown a great application potential in various clinical aspects of CRC, allowing machines to assist clinicians in many important tasks, such as colorectal polyp detection, qualitative and staging diagnosis of CRC, therapeutic assessment, as well as recurrence and survival prediction. The power of AI is poised to make practice-changing impacts on the clinical field of CRC. However, we should acknowledge that AI is still in its infancy with regard to its actual clinical application in CRC. Several challenges that must be addressed include the validation and generalizability of the clinical predictive models, the construction of interpretable models, concerns over prospective and multicenter evaluation, and the safe management and use of clinical data. We believe that in the near future, AI technologies will play a more significant role in minimally invasive screening, TNM staging prediction, and surgical treatment to further improve CRC screening, diagnosis, as well as the evaluation of treatment and prognosis.

## Figures and Tables

**Table 1:** Summary of AI applications for CRC screening. (DNN, deep neural network; SSD, single shot multibox detector; RF, random forest; LMT, logistic model trees; SVM, support vector machine; LR, logistic regression; NB, naïve Bayes; DT, decision tree; CNN, convolutional neural network; PPV, positive predictive value; Faster R-CNN, faster region-based CNN; AUC, area under the curve).

Topic	Task	Dataset	Model	Performance	Year	Ref.
CRC Screening	High-risk patient detection	111 patients’ microarray data including 22,278 features	LightGBM, DNN	Accuracy: 100%	2021	[16]
	Polyp classification	47,555 endoscopy images for 24 patients	SSD	Accuracy: 0.9067, precision: 0.9744, recall: 0.9067, F1: 0.9393	2021	[17]
	Serum biomarker detection	186 blood serum samples (39 advanced adenomas, 90 CRC and 57 healthy controls)	RF, Random Tree, LMT, SVM	Accuracy: 75%	2021	[18]
	Serum biomarker detection	263 blood serum protein samples (213 individuals undergoing screening endoscopy and 50 non-metastatic CRC)	LR, SVM, Gaussian NB, DT, RF, and extremely randomized trees	AUC: 0.75, Sensitivity: 70%, Specificity: 89%	2020	[19]
	Polyp detection and classification	27,508 endoscopy images	CNN	Detection: Sensitivity—0.92, PPV—0.86; Classification: Sensitivity—0.83, PPV—0.81	2020	[20]
	Polyp localization	EAD2019, CVC-ClinicDB, ETIS-Larib, in-house dataset, Kvasir-SEG	RetinaNet	Precision: 0.537	2020	[21]
	Polyp detection	CVC-CLINIC, ASU-Mayo Clinic, CVC-ClinicVideoDB	Faster R-CNN, SSD	Sensitivity: 0.9086, precision: 0.8154, F1: 0.8595	2020	[22]
	Polyp detection and classification	871 endoscopy images from218 patients	ResNet50, RetinaNet	F1: 0.6872, F2: 0.6607	2019	[23]
	Polyp detection	8641 endoscopy images	CNN	Sensitivity: 90.0%, Specificity: 63.3, Accuracy: 76.5%	2018	[24]
	Polyp segmentation	CVC-ColonDB	CNN	Specificity: 74.8%, Sensitivity: 99.3%, Accuracy: 97.7%	2018	[25]
	High-risk patient prediction	Colon cancer screening center data (EMRs)	Colonflag	The odds of Colonflag and normal colonoscopies: 2.0	2018	[26]
	Polyp classification	1930 NBI images	CNN	Accuracy: 85.9%, Precision: 87.3%, Recall rate: 87.6%	2017	[27]
	High-risk patient detection	112,584,133 US community-based insured data	Colonflag	AUC: 0.80 ± 0.01	2017	[28]
	High-risk patient detection	17,095 patients from KPNW (EMRs)	Mescore	Top 3% score > 97.02 Top 1% score > 99.38	2017	[29]
	Polyp detection	24 endoscopy videos	Energy map	AUC: 0.79, Sensitivity: 70.4%, Specificity: 72.4%	2016	[30]
	High-risk patient detection	606,403 Israelis and 25,613 UK dataset (EMRs)	Mescore	AUC: 0.82 ± 0.01 and 0.81 for validation sets	2016	[31]
	Polyp classification	1890 NBI endoscopic images	HuPAS version 3.1	Accuracy: 98.7%	2012	[32]

**Table 2:** Summary of AI models for CRC diagnosis and staging session. (CNN, convolutional neural network; AUC, area under the curve; SVM, support vector machine; PNN, probabilistic neural network; NL, normal mucosa; AD, adenoma; ADC, adenocarcinoma; WSI, whole slide images; RNN, recurrent neural network; TCGA, The Cancer Genome Atlas; ResNet, residual network architecture; HP, hyperplastic polyp; VGG, visual geometry group; RF, random forest; PET-CT, positron emission tomography or computed tomography; LR, logistic regression; NN, neural network; XGBoost, extreme gradient boosting; CT, computed tomography; MRI, magnetic resonance imaging; Faster R-CNN, faster region-based CNN; CAD, computer aided diagnosis).

Topic	Task	Dataset	Model	Performance	Year	Ref.
Pathological diagnosis	Tumor mutational burden-high prediction	278 HE slides	CNN	AUC: 0.934	2021	[41]
	Low/high-grade classification	Immunohistochemically stained biopsy of 67 patients	hDL-system (VGG16, SVM)	hDL-system accuracy: 99.1%; sML-system accuracy: 92.5%	2021	[42]
	NL/AD/ADC classification	4036 WSI	CNN, RNN	AUC: 0.96 for ADC; 0.99 for AD	2020	[43]
	Tumor immune microenvironment analysis	404 CRC and 20 adjacent non-tumorous tissues	CIBERSORT	C-index: stage I-II 0.69; stage III-IV 0.71; AUC: 0.67	2019	[44]
	NL/Tumor classification	94 WSI, 370 TCGA-KR, 378 TCGA-DX	ResNet18	AUC > 0.99	2019	[45]
	NL/HP/AD/ADC classification	393 WSI (12,565 patches)	CNN	Accuracy: 80%	2019	[46]
	NL/Tumor classification	57 WSI (10,280 patches)	VGG	Accuracy: 93.5%, Sensitivity: 95.1%	2018	[47]
	NL/AD/ADC classification	27 WSI (13,500 patches)	VGG16	Accuracy: 96%, Specificity: 92.8%	2018	[48]
	NL/AD/ADC classification	30 multispectral image patches	CNN	Accuracy: 99.2%	2017	[49]
	Cancer subtypes classification	717 patches	AlexNet	Accuracy: 97.5%	2017	[50]
	Polyp subtypes classification	2074 patches 936 WSI	ResNet	Accuracy: 93.0%	2017	[51]
Radiological diagnosis	Metastatic CRC prediction	MRI from 55 stage VI patients with known hepatic metastasis	RF	AUC: 0.94 (Add imaging-based heterogeneity features)	2021	[52]
	Metastatic lymph node prediction	PET-CT scan images from 199 CRC patients	LR, SVM, RF, NN, and XGBoost	AUC of LR: 0.866; AUC of XGBoost: 0.903	2021	[53]
	Colorectal liver metastasis prediction	103 metastasis samples and 80 non-cancer tissues	Probe electrospray ionization-mass spectrometry, and LR	Accuracy: 99.5%, AUC: 0.9999	2021	[54]
	Colorectal liver metastasis prediction	CT scan images from 91 patients	Bayesian-optimized RF with wrapper feature selection	AUC of radiomics features model: 86%; AUC of clinical features model: 71%; AUC of combination: 86%	2021	[55]
	KRAS mutations detection	CT scan images from 47 patients	Haralick texture analysis, SVM, LightGBM, NN, and RF	Accuracy: 83%, kappa: 64.7%	2020	[56]
	Classification of T2 and T3	290 MRI images from 133 patients	CNN	Accuracy: 0.94	2019	[57]
	Metastatic lymph node prediction	MRI images from 414 patients	Faster R-CNN	r-radiologist-Faster R-CNN 0.912	2019	[58]
	Polyp detection	825 CT scan images	CNN	Accuracy: 0.87, Sensitivity: 0.8877, Specificity: 0.8735	2017	[59]
	Polyp detection	154 CT scan images	CNN	Accuracy: 0.971	2017	[60]
	Polyp classification	1035 endomicroscopy images	Mathworks “NAVICAD” system	Accuracy: 84.5%	2016	[61]
	Polyp detection and classification	148 CT scan images	Haralick texture analysis, SVM	ROC: 0.85	2014	[62]
	CAD system for polyp detection	24 T1 stage patients’ CT scan images	Coloncad API 4.0, Medicsight plc	True positives rate >96.1%	2008	[63]

**Table 3:** Summary of AI applications for CRC treatment session (nCRT, neoadjuvant radiotherapy; ANN, artificial neural network; AUC, area under curve; KNN, K-nearest neighbors; SVM, support vector machine; NBC, naïve Bayesian classifier; MLR, mixed logistic regression; LR, logistic regression; NN, neural network; BN, Bayesian network; RF, random forest; CPT-11, Irinotecan; IC50, half maximal inhibitory concentration).

Topic	Task	Dataset	Model	Performance	Year	Ref.
nCRT	nCRT response prediction	Medical records from 282 patients (248 training and 34 validation)	ANN, KNN, SVM, NBC, MLR	ANN model outperformed others: Accuracy: 0.88, AUC: 0.84, Sensitivity: 0.94	2020	[79]
	nCRT response prediction	6555 patients’ records from the SEER	LR	3-year OS rate: 92.4% with pCR; 88.2% without pCR	2019	[80]
	nCRT response prediction	98 patients MRI (53 training set and 45 validation set)	SVM, NN, BN, KNN	Test: AUC: 97.8%, Accuracy: 92.8%, Validation: AUC: 95%, Accuracy: 90%	2019	[81]
	nCRT response prediction	55 patients MRI	RF	Mean AUC: 0.83	2019	[82]
Chemotherapy	The toxicity of CPT-11 prediction	Demographic data, liver function bloody tests and tumor markers from 20 advanced CRC patients	SVM	Accuracy: 91% for diarrhea, 76% for leukopenia, and 75% for neutropenia	2019	[83]
	Drug IC50 detection	18,850 organic compounds	KNN, RF, SVM	Accuracy: over 63%	2018	[84]

**Table 4:** Summary of AI models for CRC prognosis session. (C-index, concordance index; LR, logistic regression; DT, decision tree; GB, gradient boosting; LightGBM, light gradient boosting machine; CNN, convolutional neural network; AUC, area under curve; PET-CT, positron emission tomography or computed tomography; HR, hazard ratio; GSEA, gene set enrichment analysis; PPI, protein-protein interaction; HE, hematoxylin and eosin; WSI, whole slide image; MLP, multilayer perceptron; AdaBoost, adaptive boosting; LSTM, long short-term memory; EHR, electronic health record; SVM, support vector machine; NB, naïve Bayesian; KNN, K-nearest neighbors; NN, neural network; RF, random forest).

Topic	Task	Dataset	Model	Performance	Year	Ref.
Recurrence	Recurrence perdition of stage II CRC	Clinicopathological data of 350 patients after curative resection for stage II CRC	Nomogram	C-index: 0.585 in the validation set	2020	[88]
	Recurrence prediction of Stage IV CRC after tumor resection	EHR data from 999 patients of stage IV CRC	LR, DT, GB and LightGBM	LightGBM: AUC: 0.761	2020	[89]
	Recurrence prediction of local tumor	PET-CT images from 84 patients	CNN, Proportional hazards model	C-index: 0.64	2019	[90]
	Risk prediction of recurrence of gastrointestinal stromal tumor	Clinical data of 2560 patients	Proportional hazards, Non-linear model	AUC: 0.88	2012	[91]
	Recurrence perdition after surgery	Clinicopathological data of 1320 nonmetastatic CRC patients	NomogramCOX regression	C-index: 0.77	2008	[92]
Survival	Genetic risk factors Identification	National Center for Biotechnology Information Gene Expression Omnibus	GSEA, PPI network, Cox Proportional Hazard regression	4 sub-networks and 8 hub genes as potential therapeutic targets	2021	[93]
	Prognostic prediction for stage III CRC	Clinicopathological data of 215 patients	CNN, GB	HR: 8.976 and 10.273	2020	[94]
	Outcome prediction	12,000,000 HE images	CNN	HR: 3.84 and 3.04 with established prognostic markers	2020	[95]
	Survival prediction	7180 HE images of 25 patients	CNN	Nine-class accuracy: >94%	2019	[96]
	Survival prediction	PET-CT images of 84 patients	CNN, proportional hazards model	C-index: 0.64	2019	[90]
	Outcome prediction, and remaining lifespan prediction	SEER	tree-based ensemble model	Accuracy: 0.7069, Sensitivity: 0.8452, Specificity: 0.66	2019	[97]
	Outcome prediction	75 WSIs from stage I and II CRC patients with surgical resection	CNN	F1: 0.67	2019	[98]
	Outcome prediction	EHR data of 58,152 patients	CNN	AUC: 0.922, Sensitivity: 0.837, specificity: 0.867, PPV: 0.532	2019	[99]
	Prediction of Stages and Survival Period	Clinicopathological data of 4021 patients	RF, SVM, LR, MLP, KNN, and AdaBoost	RF: F-measure: 0.89, Accuracy: 84%, AUC: 0.82 ± 0.10	2019	[100]
	1/2/5 years Survival prediction	SEER data	DNN	AUC: 0.87	2019	[101]
	Outcome prediction	Digitized HE tumor tissue microarray samples of 420 patients	CNN, LSTM	LSTM: AUC: 0.69, histological grade AUC: 0.57, the visual risk score AUC: 0.58	2018	[102]
	5-year survival prediction	EHR data of 1127 CRC patients	Ensemble (bagging and voting) classifier	Ensemble voting model AUC: 0.96	2017	[103]
	5-year survival prediction	EHR data of 334,583 cases from Robert Koch Institute	SVM, LR, NB, DT, KNN, LR, NN, RF	Average accuracy of the clinicians: 59%, ML: 67.7%	2015	[104]
